# Metals detected by ICP/MS in wound tissue of war injuries without fragments in Gaza

**DOI:** 10.1186/1472-698X-10-17

**Published:** 2010-06-25

**Authors:** Sobhi Skaik, Nafiz Abu-Shaban, Nasser Abu-Shaban, Mario Barbieri, Maurizio Barbieri, Umberto Giani, Paola Manduca

**Affiliations:** 1 Shifa Hospital, Gaza, Palestine; 2 Plastic surgery Dept. and Burn Unit, Shifa Hospital, Gaza, Palestine; 3 FRCSI, Gaza, Palestine; 4 Istituto di Geologia Ambientale e Geoingegneria, C.N.R., Rome, Italy; 5 Dept. Scienze della Terra, University of Rome, Rome, Italy; 6 Dept. of Preventive Medical Sciences, Faculty of Medicine, University Federico II, Naples, Italy; 7 Dept Biology, University of Genoa, Genoa, Italy

## Abstract

**Background:**

The amount and identity of metals incorporated into "weapons without fragments" remain undisclosed to health personnel. This poses a long-term risk of assumption and contributes to additional hazards for victims because of increased difficulties with clinical management. We assessed if there was evidence that metals are embedded in "wounds without fragments" of victims of the Israeli military operations in Gaza in 2006 and 2009.

**Methods:**

Biopsies of "wounds without fragments" from clinically classified injuries, amputation (A), charred (C), burns (B), multiple piercing wounds by White Phosphorus (WP) (M), were analyzed by ICP/MS for content in 32 metals.

**Results:**

Toxic and carcinogenic metals were detected in folds over control tissues in wound tissues from all injuries: in A and C wounds (Al, Ti, Cu, Sr, Ba, Co, Hg, V, Cs and Sn), in M wounds (Al, Ti, Cu, Sr, Ba, Co and Hg) and in B wounds (Co, Hg, Cs, and Sn); Pb and U in wounds of all classes; B, As, Mn, Rb, Cd, Cr, Zn in wounds of all classes, but M; Ni was in wounds of class A. Kind and amounts of metals correlate with clinical classification of injuries, exposing a specific metal signature, similar for 2006 and 2009 samples.

**Conclusions:**

The presence of toxic and carcinogenic metals in wound tissue is indicative of the presence in weapon inducing the injury. Metal contamination of wounds carries unknown long term risks for survivors, and can imply effects on populations from environmental contamination. We discuss remediation strategies, and believe that these data suggest the need for epidemiological and environmental surveys.

## Background

In 2006 and 2009 at Shifa Hospital in Gaza we received victims with limb amputations, bodies completely charred in open air, and in 2009, people with more superficial burns and with deep burns containing self-igniting material, the last due to white phosphorus (WP) [1]. In all these victims, no fragments of weapons could be recovered by surgical inspection, X rays or ecografy.

Similar injuries were previously reported to occur with high frequency in other recent wars.

The use of metals and heavy metals in weapons is implicated as the cause of the injuries without fragments; it is an outstanding question whether the toxic and genotoxic potential of metals used in weapons could be a cause for long-term health damage in exposed populations and the military.

Information accumulated in the last 6 years in Iraq shows an increase in oncologic, chronic and reproductive diseases and in malformations at birth, particularly high in the areas most severely attacked [2,3]. Syndromes of various types, including deformities in progeny, have also been reported in military and paramilitary personnel returning from this war.

In the specific literature, the "enhancement" of already existing weapons of war by the utilization of particulate and potentially toxic metals has been described. This has led to the commissioning of weapons utilizing metals as augmenters, or as primary effective agents (small smart bombs, thermobaric grenades and shape charged weapons, these latter being able to produce a 'molecular sieve' of metal powder, capable of severing the human body) [4-6]. The timing of deployment of these items is compatible with their use before 2006 also in Gaza and Lebanon.

The health related issues raised by these circumstances are many and complex.

Here we investigated the association of metals with wound tissues from different classes of fragment-free injuries from victims of military operations in Gaza in 2006 and 2009.

These were classified in the emergency rooms in classes, according to the injury; biopsies were collected at the site of wounds to undertake their analysis for content of 32 metals by ICP/MS. The ICP/MS analytical procedure is appropriate for metal detection in biologic samples [7] and is used in studies of accumulation of metals in tumors [8] and in body fluids, in clinical, epidemiological and experimental investigations of biological materials [9,10].

Biopsies were obtained from surviving and dead individuals with amputations (A), from dead individuals with charred bodies (C), from surviving individual with deep burns with self-igniting material, by WP (M), and from individuals with simple burns (B), and, for control, from individuals without injuries.

We here report that among the metals that we detected in excess over control in the biopsies at wound sites for all classes of injuries there are different combinations of known human carcinogenic/teratogenic metals (class 1, IARC) as Hg, As, Cd, Cr, Ni [11], and U (A1, Confirmed Human carcinogen by ACGIH and NIOSH), of possible carcinogens (class 2B, IARC) as Co, V, and of known fetotoxic metals as Al, Cu, Ba, Pb, Mn [12-14]. Toxicological, epidemiological and experimental studies [12-15] established that excess of these metals can disrupt body functions and have pathogenic effects in human respiratory organs, kidney and skin and affect sexual and neurological development and functions. Excess metal assumption in experimental animals [16-22] and in vitro [23] interferes with the oxidative/redox pathways, with hormone receptor mediated transcription, and, directly and indirectly, affects DNA integrity, repair and transcription [24-26].

## Methods

Victims were classified according to injury at entrance in Shifa Hospital and recorded in photos taken by Doctors (not shown). The biopsies for analysis were taken with the oral consent of the live ones at surgery/medication or at death, from the site where injury broke the body continuity and included the skin and dermis whenever possible. In some cases, bone exposed by the wound was also collected. All biopsies were preserved immediately in formaldehyde and stored refrigerated. Approval for this procedure was obtained from the Ministry of Health of the Palestinian Government. Control biopsies of mm of skin and dermis were obtained from two healthy consenting individuals undergoing femur replacement surgery, and a sample of trabecular bone from the medullar channel dug to apply the prosthesis from one of them (donated with consent; courtesy of Prof. A. Federici, Recco Hospital, Italy). These were used as reference controls in analysis respectively for skin/derma and for bone biopsies of victims. All samples were dissected, on arrival in the central lab, by the same operator, perpendicular from the surface of the skin towards the derma for all the biopsies from victims with amputations and controls, unless stated differently, and when dermis was present, also for burn patients. Fragments of about 0,04-0,07 g wet weight were obtained from each biopsy, recoded, set in the same batch of 3% PAF, pH 7,2 and delivered, blinded, to each of 3 analytical laboratories. With the exception of two very small biopsies, for all others between two and seven different fragments were analyzed in at least two, of three different laboratories.

Tissue classification was done under stereo-microscopy and for some of the samples (G1, G2, R1, R2, N5 and N7) was confirmed by histological analysis of sections from paraffin sections (H.E., Masson and DAPI stain, courtesy of A. Strangio, DIMES, University of Genoa, Italy).

Preliminarily, aliquots of the PAF solution and of the original formalin in which biopsies were preserved were analyzed by ICP/MS to rule out background from fixatives. Controls from normal tissues were included in duplicate or triplicate in each run. Digestion and processing were in each analytical laboratory, according to the procedures below: Lab *a *at the AUB, Beirut, Lebanon*: *Samples digestion was in 1 M HNO3; multiple runs were performed with different dilutions (1/1000,1/100 and 1/10) of the digest. Calibration curve was in nitric acid 70% (Fisher) with calibration standards (Agilent). Verification standards were from Absolute Standards. Analyzer was ICP-MS model 7500 Ce (Agilent). Lab *b *at Chalmers University, Goteborg, Sweden: Samples digestion was 1 M HNO3 containing internal standards (Mercks). Analyzer was Elan 6000 (PerkinElmer). Lab *c *at the University of Rome, Italy: Samples digestion was in H2SO4, HNO3. Standards (Carlo Erba, Italy) and control reference materials were run in parallel with samples. Analyzer was XSERIES 2 ICP-MS Thermo. In all laboratories, the analysis of a single run on the ICP-MS was the average of 3 uptakes by the instrument. Values of metal content in the normal tissue of control obtained in each lab and each set of runs are expressed in ppb and reported at the bottom of Table 1. The values of the metal content in each biopsy from the victims were divided by the corresponding control (same tissue and same analytical run) and are reported as folds over control in the Tables. Al, Ti, Cu, Sr, Ba, Co, Mo, W, U, Cr, Li, B, Mn, Rb, As, Cs, Hg, Zn, Ni, Sn, Pb, V, Cd, Ga, Be, Pt, Tl Mg, Na, K, Ca, and Sb were analyzed, utilizing the most frequent isotope as standard.

**Table 1:** Fold over control of Metal content in biopsies - ICP/MS analysis

	°^		°^	^	°?	/	*	/§		°			*	°^			*	*§		*	/§			
																								

**Sample**	**Al**	**Ti**	**Cu**	**Sr**	**Ba**	**Co**	**Hg**	**V**	**Sn**	**Pb**	**B**	**LI**	**As**	**Mn**	**Rb**	**Cs**	**Cd**	**Cr**	**Zn**	**U**	**Ni**	**tissue**	**class**	***Lab***

**N 1**	1,8	0,8				3,5			1	**100**										3.55	0,6	skin	B	*b*

**N 2**	0,76	1,47				1,4		nd	0,09	1,2										1.3	0,25	skin	B	*b*

**N2**	0,97	nd	0,66	0,47	0,67	0,4	8,5		4	2,1	0,76	3	8,7	2,3	4	3,5	2,6	3,1	1,2		3	skin	B	*c*

**N 15**	1,4	nd	1,1	0,02	nd		14	nd	nd	nd	nd	nd	nd	nd	nd	nd	nd	1,2	3		1,1	skin	B	*c*

**N15**	3,6	nd	2,1	0,009	nd		0,35	nd	nd	nd	nd	nd	nd	nd	nd	nd	nd	0,81	1,4	3	0,9	skin surface layer	B	*c*

**N15**	1,9	0,45							nd	0,29										4,4	nd	skin	B	*b*

																								

**G1**	2,1		1,9	0,3	1,1	0,2	8,5	18,5	nd	0,17	1,1	0,27	6,2	0,6	5,2	0,25	nd	0,07	2,7	0,33	0,17	muscle	B/C	*c*

**G1**	**36,9**	0,8	2,3	0,45	0,6	0,9			0,87									0,33			0.009	muscle	B/C	*a*

**G1**	7.98	0,162	0,48			2,5			1,1	7										7,1	0,9	muscle	B/C	*b*

																								

N12**	**1564**	16				nd			nd	9,5										7,4	nd	skin to muscle	M	*b*

N12**	1,8	nd	1,1	0,02	nd	nd	nd	nd	0,65	0,21	0,5	0,5	0,25	0,27	0,3	0,3	0,3	0,23	1,8	1,6	0,2	skinwhole	M	*c*

N12**	7,1	nd	5,6	10,6	6,2	9,3	4,3	nd	nd	0,04	nd	nd	nd	nd	nd	nd	nd	1,5	1,7		1,4	skinwhole	M	*c*

N13**	**184,1**	nd	8,8	2,8	6,4	3,2	nd	nd	0,3	0,21	0,1	0,5	nd	0,8	0,3	0,4	0,3	0,4	0,28		0,22	skin surface layer	M	*c*

																								

**N 3**	**59,5**	**56,4**				12,1			7,93	**20**										2,75	2,5	skin	B/T	*b*

																								

**R2**	**73,8**	14,9	48,2	14,9	**47,9**	1,3			2									0,73			0,05	lamellar bone	C	*a*

**R2**	**102**	**90**				9,3			3,3	**42**										1,83	1,6	lamellar bone	C	*b*

**R2**	39		43	5,6	12	1,7	13,9	**277**	nd	0,15	**44,5**	0,7	>15	3,45	9	1,8	nd	0,32	3,3	3,56	0,5	lamellar bone	C	*c*

N9*	15	**330**				1,4			0,7	3,5								0,77		6,3	1,98	soft tissue	C	*b*

N9*	4,4	nd	3,8	11	15	5,3	**26**	2,2	0,9	0,38	3	0,57	1,2	0,7	2,3	0,9	nd	1	2,9	1,7	0,25	unburned bone	C	*c*

N10*	2,8	nd	0,75	6,9	3	2,2	17,8	2	1,6	0,5	4,7	1	>5	1	2,6	2,1	nd	0,8	2,8	4,49	1,1	soft tissue	C	*c*

N10*	**84**	**218**				7,7			4,2	8,6										1	3,7	skin	C	*b*

N11*	21	8,7	1,1	**208**	7	1			1,5									0,04			0,02	lamellar bone	C	*a*

																								

**N4**	**356**	17,3				1			**60,5**	15,8										8	2	skin to muscle	A	*b*

**N4**	11,4	13,81	**38,4**	17,3	**20**	1,2			1,3									0,25			0,04	skin to muscle	A	*a*

**N5**	**31,9**	7,3	**38,9**	15,7	11,6	1			3,5									0,61			0,07	skin surface layer	A	*a*

**N5**	**47,7**	9,5				2,1			12,2	15,2										0,25	1	skin surface layer	A	*b*

**N5**	**82,1**	nd	**51,1**	7,9	7,3	**58,3**	**34**	**130**	<0,01	0,01	nd	nd	**28**	nd	nd	nd	nd	8,7	3		6,5	skin surface layer	A	*c*

**N5**	19,3	nd	2,3	6,6	0,8	11,1	10,5	92,5	**370**	4,3	24,3	9,6	7,3	3,4	7,1	4	3,9	3,6	150		12,5	skin surface layer	A	*c*

**N6**	**2355**	**815**				6,3			44,5	18										20	8,4	skin to muscle	A	*b*

**N6**	**24,9**	nd	47	0,01	nd	nd	9	**130**	nd	nd	nd	nd	nd	0,03	nd	nd	nd	2,5	6,4	2,66	2,2	derma	A	*c*

**N7**	22	nd	42	0,34	nd	**21,6**	35	**122**	**357**	3,6	2,1	**22,7**	9,4	2	5	2	49,2	4,7	8,5		12,5	skin surface layer	A	*c*

**N7**	7,8	nd	3,3	4,6	nd	1	nd	nd	12,2	2,9	2,5	1,5	1,5	2,5	4,1	1,8	>6	0,41	1,2		0,41	lamellar bone	A	*c*

**N7**	1,5	nd	2,1	2,35	nd	1	nd	nd	15,8	7,9	5	1,9	nd	3,2	7,4	3,9	<10	0,84	0,81		0,74	trabecular bone	A	*c*

**N7**	**31,9**	6	**108,2**	8,5	18,5	9,5			**59,6**									0,93			0,37	soft tissue	A	*a*

**N7**	**1200**	32,5				1,87			**2155**	**78,2**										1	5	bone marrow	A	*b*

**N8**	6,4	nd	1,6	2,9	8,9	2,3	18	**50**	1,1	0,9	0,6	2,1	10,7	2,2	3,8	1,7	1,2	1,4	2,8		1,5	skin	A	*c*

**N8**	4,6	nd	1,3	2,47	5,8	1,5	nd	**25,3**	0,5	0,31	0,37	1,7	nd	1,5	3,6	1,2	0,4	0,46	1,5		0,41	skin	A	*c*

**N8**	6,6	nd	1,5	4,5	11,4	2,3	0,6	**29,6**	1,35	0,9	0,8	2,3	1,7	1,6	0,6	1,9	1,1	1,3	3,3		1,1	derma	A	*c*

**N8**	**553**	88				0.66			9,3	7,33										6,6	1,22	skin to muscle	A	*b*

**G2**	10,27	15,2				3,7			14,35	nd										nd	0,4	skin to muscle	A	*b*

**G2**	**71,8**	18,2	**59,8**	13,9	**70**	2,5			10									0,6			0,12	skin to muscle	A	*a*

**G2**	**83**		5,7	0,5	3,6	1	**53**	**49**			2,9	2	12,5	0,22	7,9	0,8	nd	0,14		2,7		skin to muscle	A	*c*

**R1**	9,8	11,8				4,6					2,3	1	9,4	1,2	1,5	0,3		0,11		0,5		muscle	A	*b*

**R1**	**30,3**		6	1,5	2,2	0,5	**71**	**46,3**	**37,7**									0,93		5,37	0,12	muscle	A	*c*

**R1**	**73,7**	16	**177**	**67**	**91,6**	3,3			4,5									0,33		2,39	0,12	soft tissue	A	*a*

**R1**	**23,2**	10	**44,1**	**22,7**	**22,9**	1,5																		

																								

**Sample**	**Al**	**Ti**	**Cu**	**Sr**	**Ba**	**Co**	**Hg**	**V**	**Sn**	**Pb**	**B**	**Li**	**As**	**Mn**	**Rb**	**Cs**	**Cd**	**Cr**	**Zn**	**U**	**Ni**			

**ppb**																								
**1**	3900	645	2248	1021	281	234			1129									7130		93	26350	skin	healthy	*b*

**1**	11300		1800	3390	1000	69	5,25	<1	581	6300	145	13	7,6	3587	11	16,8	0,047	950	4672	22,4	1797	skin	healthy	a*

**1**	7300		400	662	250	20	0,33	10,9	271	1690	1780	59	<1	2168	12	27	0,188	3120	8043		4205	skin	healthy	c*

**2**	25300		2700	3400	1223	16	<1	54	912	1000	560	177	34	530	37	11	0,289	6600	8301		4207	skin	healthy	c*

**3**	26300		1500	9000	1134	38	2		271	1300	335	133	<1	2156	26	12,5	nd	8500	34210		5415	skin	healthy	c*

nd is for not detectable and in bold are the values over 20 folds that of the average of the controls run in the same laboratory; for triplicate runs of each sample σ was always less than 5% and is not shown. In the first column, * and ** are fragments from biopsies in different wounded sites from the same victim. In the last column, * indicates that each value is the average of 2 or more runs. On the top, ° fetotoxic, ^pathogenic, *carcinogenic class 1 IARC,/possible carcinogenic class 2B, IARC, §only in some forms.

More than one fragment was dissected from each biopsy, digested and analyzed blind (each in triplicate runs), in 3 different laboratories (on the far right labeled as *a, b *and *c*). Samples from each victim are identified on the left by a letter in upper case and a number, corresponding to those described in Table 2. On the right side, in sequence, the identification of the kind of tissue for each fragment, the class of injury (represented by a case letter: A-amputation; B-burns; C-charred; M-multiple piercing burns by WP; B/T and B/C unclassified). Below are the values in ppb of the controls for each run and each lab; the number at the left identifies the biopsies. Modality utilized for calculation of folds are described in M&;M.

Top row, above the metal names, the symbols refer to their classification as detailed at the bottom of the Table. A code color is used to visualize different increase in folds/control. Asterisks identify multiple biopsies from different anatomical sites of a same individual.

The Mann-Whitney U test was applied to identify significance of the differences in metal concentrations among the most numerous categories of injuries, i.e. A, B and C.

## Results

We analyzed fragments dissected from 18 biopsies derived from 15 victims of war-derived injuries, in 2006 and 2009. Injuries were classified by doctors according to clinical criteria (Table 2). We analyzed biopsies obtained from seven victims with amputations (A) in 2006 and 2009, three with burns (B) in 2009, two charred bodies (C) one in 2006 and one in 2009, and one case of multiple piercing burns (M) with self igniting material (WP) in them in 2009, plus two samples, tentatively classified B/C (severely burned/partly charred) in 2006 and B/T (a victim with burns accompanied by extensive internal edema, suggestive of the effect of a wave of pressure blast) in 2009. In all classes of accident some kind of explosive burst was involved and no fragments were detected even with Xrays. For two victims more than one biopsy was taken, from different wounds, and asterisks in Table 1 refer to these.

**Table 2:** Clinical classification for type of wound and description of each victim were done at the arrival at the Hospital, and biopsies were grouped accordingly.

Classification of biopsies from different type of wounds and clinical observations
**A -**mono or bilateral amputations by weapon of lower limbs throughout the bone, showing sharp rescission or with shredded flesh. Often presenting also punctuated round holes in the pubis. No metal fragments detectable by X rays or by surgical inspection. Biopsies taken from the rim of the amputated limb, inclusive of skin to muscle, and sometimes bone fragments.



**B - **burn injuries, of different extend and depth. Biopsy were from the burnt skin.



**C -d**eeply burned bodies, charred to the bone. Biopsies from the muscle underneath the burn and from the exposed burnt lamellar bone.



**M-**multiple and diffuse burn injuries with roundish shape and devoid of snarpel at inspection. Self ingniting clumps, of White phosphorus, were extracted from the place of the wounds.Biopsies included the rim of different wounds.



**B/T - **suspected injury by pressure wave; patient with diffuse burns and overlay of grayish material, with internal edemas. Biopsy was from the burnt skin.



**B/C **- severely and diffusely burn injuries, charred sections of the body. Biopsy was from the muscle underneath the burn.



**Sample Number/Class of injury/Description**

**1-B- **Explosive injury on 5/1/2009 with deep burns\necrosis of chest wall skin, muscle and bone of sternum. Biopsy on 14/1/2009.

**2-B- **Explosive injury on 12/1/2009 with burning of face, chest wall and limbs. Biopsy on 15/1/2009.

**3-B/T **Explosive injury on 12/1/2009 with second and third degree burns both hands with a layer of gray material on top of burn. Biopsy on 14/1/2009.

**4-A **Explosive injury on 14/1/2009 with crushed and nearly amputated both lower limbs. Biopsy from skin and muscle of Rt lower limb. Biopsy at surgery.

**5-A- **Explosive injury on 15/1/2009 with crushed lower limbs. Biopsy on 15/1/2009.

**6-A- **Explosive injury on 14/1/2009 with crushed Rt arm. Biopsy on 14/1/2009.

**7-A- **Explosive injury on 15/1/2009 with crushed lower limb. Biopsy on 15/1/2009

**8-A- **Explosive injury on 16/1/2009 with both lower limbs crushed. Biopsy on 16/1/2009.

9*-C- Explosive injury on 15/1/2009. Completely burned body. Biopsy on 15/1/2009. burned derma.

10*-C- Same victim as in 9. Soft tissue.

11 *-C- Same victim as in 9. Carbonized bone marrow.

12**-M- Injury by self igniting material on 11/1/2009, with multiple areas of deep burn and necrosis of skin, subcutaneous tissue and muscle. Biopsy on 14/1/2009.

13**-M- Same victim as in 12. Subcutaneous tissue and muscle. Biopsy on 14/1/2009 from muscle.

**15-B- **Explosive injury on 11/1/2009 with areas of deep burn on Rt thigh. Biopsy at recovery from necrotic skin of right thigh.

**G1-B/C- **July 2006. Severe burns covering the whole body. Biopsy at the recovery.

**G2-A- **July 2006. Amputation of lower limb. Biopsy at the recovery.

**R1-A- **July 2006. Amputation of lower limb. Biopsy at the recovery.

**R2-C- **July 2006. Completely charred body, charred cranial bones. Biopsy at the recovery.

In Table 1 the samples analyzed are listed according to the class of injury (as defined in Table 2). Results of ICP/MS determinations are presented as folds over control biopsies from normal tissues examined in the same run. Analysis was done for 32 elements. Some elements were detected in all biopsies in similarly high amounts (Mg, K, Na), some were detected in similarly low amounts as in control (Ca, Sb, In), and not all elements were detectable in control and samples (Ga, Mo, W, Be, Tl); all these above are not reported in Table 1, which therefore includes 21elements.

With regard to the presence of the metals, Table 1 shows that there was presence above control of many metals in the tissues from all the victims, including known carcinogenic and toxic metals and associations of these: Hg and U were found in biopsies of all classes, As in biopsies from A, B and C victims, Cd and Cr in biopsies from A and B victims.

Presence of possible carcinogens (Co, V, Ni) and fetotoxic (Al, Mn, Pb, Cu and Ba) metals further characterized each class of wounds: these were all present in low (> 1 to 3 folds of control) to high (> 10 folds of control) amounts in class A; V did not exceeded control in class M, and Ni did not exceeded control in class C. We used in Table 1 a color code to facilitate visualization of the excess in folds over control.

Of the other metals reported in Table 1, Rb, Zn, and Cs were detected in different amounts over the control, in biopsies of classes A, B and C, not in M; B was over control in A and C; Li in biopsies of classes A and B; Sn and Pb were constantly detected in all classes; Sr, Ti, Cu and Ba were not detected in biopsies of class B.

The potential metal load from the amounts of these metals was: high amounts (> 10 folds of control) for Al, Ti, Ba, V and for the carcinogenic Hg, were consistently in fragments from A biopsies; High amounts of Al, Ti, Sr, Ba and Hg in class C, and of Al and Ti in class M biopsies. None of these was detected at similar high level in biopsies from B injuries.

These data show that there is a toxic and carcinogenic load for each injury by weapon, that this varies in kind and amount for different classes of injuries, and in all cases it consist in co-presence of more metals.

**Figure 1:**
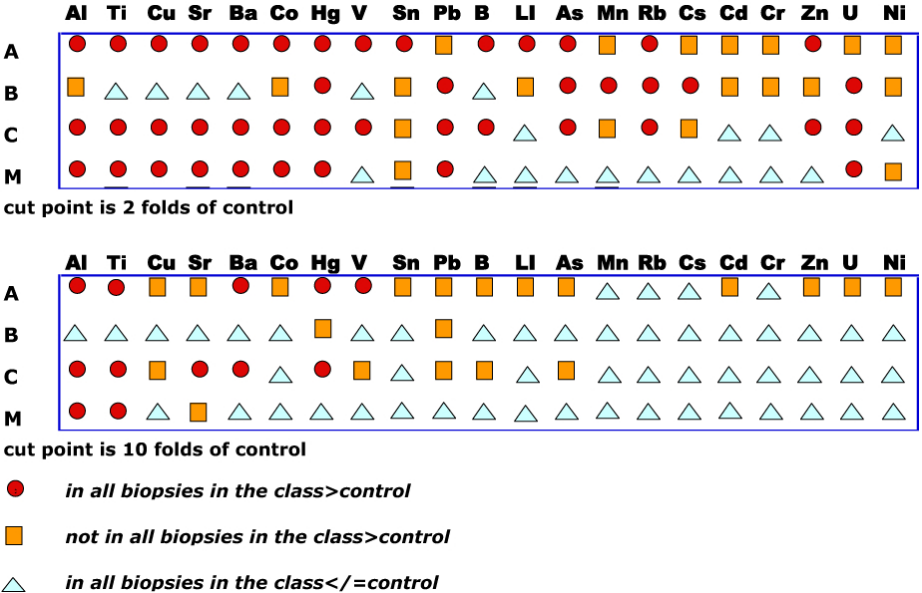
**Graphic summary of the metal presence in wounded tissues**. A- The symbols indicate the detection of metals in amount higher than 2 folds than in control. B- The symbols indicate the detection of metals in amount higher than 2 folds than in control. On the left: A for amputation, B for burned, C for charred, M for multiple piercing burns. Circles- Metals are detected in all the biopsies in a class of injury in amounts above the cut point, below in the Figure. Squares- Metals are detected only in some biopsies of a class of injury in amounts above the cut point. Triangles- Metals are detected all biopsies of a class of injury in amounts below the cut point.

For the most numerous injuries analyzed, (A, B, C) and that from which more than one biopsy was taken in different wounds (M), we derived a graphic description of the distribution of relevant metals (Fig. 1): in Fig. 1A, we show that all biopsies of a class present a characterizing "metal signature", whereby they have equal or more than 2 fold amount of specific metals. This signature varies for each class of injury and points to the specific relationship between class of injury and type of metals delivered by the weapon causing it. In Fig. 1B, using a 10fold amount as limit, is shown that the amount of each metal in the different classes of injuries differs. Together, these Figures describe semi-quantitatively the specific metal signature associated to each kind of injury.

Although we analyzed many fragments from each biopsy, the limited number of total biopsies available allowed only limited statistical treatment aimed to compare the clinical classes of injuries and the amounts in metals content. Application of the test of Mann-Whitney to compare metal concentrations between the categories of injuries A, B and C, showed a statistically significant (p < 0.05) higher concentration of Al and Hg in class A with respect to class B (Table 3), confirming a specific metal signature of the amputating weapon.

**Table 3:** Significance of the differences in amount of metals

Comparison of category A vs category B	
**Metal**	**P value**
Al	0,017
Ti	0,521
Cu	0,079
Sr	0,143
Ba	0,234
Co	0,77
Hg	0,046
V	0,134
Sn	0,243
As	0,329
Mn	0,699
Rb	0,329
Cs	0,845
Cd	0,688
Cr	1
Zn	0,064
Ni	0,795

Significance of the differences in amount of metals between classes A and B was evaluated by Mann Whitney test. p < 0,05 is significant difference.

Comparison of the results from analysis of the samples (Table 1) from 2006 (two A and two C biopsies) and those from 2009 (seven A and two C biopsies), suggests that the metal signature described here for each class of injuries has remained similar in time for similar accidents.

The clinically unclassified biopsies B/C and B/T, show a pattern of metal content different from each other and from the other classes. This apparently agrees with the existence a specific metal signature-injury relationship.

We also analyzed by ICP/MS the distribution of metals for three A and one C biopsy dissecting the biopsy in a way to purposefully separate tissues located further the body from the site of direct exposure to the injuring weapon from those directly exposed (Table 4). The biopsies were either dissected in order to select fragments of tissues at progressive depth from the surface of the skin (N5 and N7), or to select exposed muscle and underlying not exposed soft tissues (R1), or to select weapon impacted bone versus not impacted muscle (R2). Metals were found in a decreasing gradient from the skin to the underlying tissues, and from carbonized bone versus muscle not carbonized. This indicates that the metals concentration is inversely related to the distance from the point of "hit", regardless if this is the outside skin or the exposed internal bone, and confirms that metals were delivered by the weapon, supporting the validity of our approach of measuring metals at the wound site to obtain proof of their deliverance by weapons. In addition, these data help to rationalize a source of variability (see Table 1) in the absolute amounts of metals we found in analyzing different fragments similarly derived from the same biopsy; they agree with the possibility that the above variability is due to differences in the depth of the perpendicular skin-dermis fragments utilized in different determinations from the same biopsy.

**Table 4:** Fold over control of Metal content in tissues at a different distance from the site impacted by the weapon

Sample-tissue	Al	Ti	Cu	Sr	Ba	Cr	class	*Laboratory*
**N5 **-surface skin	**47,7**	9,5					**A**	***a***
**N5 -**skin to fat	7,9	2,21						***a***
**N5 -**fat layer	1,2	0,89						***a***
								
**N5 -**surface skin	14,3	1,3	9,08	3,61	2,68	0,1	**A**	***b***
**N5 -**skin to fat	1,79	0,74	0,11	2,7	1,1	0,2		***b***
**N5 -**fat layer	3,29	0,96	0,11	1,11	1,32	0,2		***b***
								
**N5 -**surface skin	**159**						**A**	***b***
**N5 -**skin to fat	**44**							***b***
**N5 -**fat layer	2,8							***b***
								
**N5 -**surface skin	**82,1**		**51,1**	7,9	7,3	1	**A**	***c***
**N5 -**skin-dermis	19,3		2,3	6,6	0,8	0,6		***c***
**N5 -**fat layer	4,6		2	nd	nd	1		***c***
								
	**Al**	**Ti**	**Cu**	**Sr**	**Ba**	**Cr**		
**N7-**skin surface layer	**22**	nd	**42**	0,34	**nd**	0,96	**A**	***c***
**N7-**lamellar bone	7,8	nd	3,3	4,6	nd	0,5		***c***
**N7- **trabecular bone	1,5	nd	2,1	2,35	nd	0,5		***c***
								
	**Al**	**Ti**	**Cu**	**Sr**	**Ba**	**Cr**		
**R1 -**muscle	9,8	11,8					**A**	***a***
**R1 -**soft tissue	0,63	2,3						***a***
								
**R1 **-muscle	14,1	8,66	34,2	13	13	0,2	**A**	***b***
**R1 **-soft tissue	4,9	1,98	9,43	4,85	4,89	0,1		***b***
								
	**Al**	**Ti**	**Cu**	**Sr**	**Ba**	**Cr**		
**R2 -**bone	**102**	**90**					**C**	***a***
**R2 **-muscle	4,3	7,8						***a***
								
**R 2 **-bone	**41,7**	8,3	**48,2**	14,9	**26.89**	0,4	**C**	***b***
**R2 **-muscle	1,45	1,51	2,82	5,26	2,89	4,5		***b***

blank box= not done; nd below detection; Bold the values above 20 folds of control values.

All the biopsies analyzed here were from patients killed as consequence of the injury they received. The biopsies were dissected purposefully in order to select fragments of tissues at progressive depth from the point of impact of the weapon: for N5 is compared metal content in the surface of the wounded skin to that in deeper tissue layers not directly severed by impact; for N7 is compared metal content in the surface of the wounded skin with that of exposed lamellar bone and than of more internal trabecular bone; for R1 is compared metal content in muscle exposed by the wound with underlying soft tissue; for R2 is compared metal content in blackened bone with not burned muscle. Similarly prepared fragments of N5 were analyzed in 3 different laboratories, those of N7, R1 and R2 in 2 laboratories.

## Discussion

We investigated if metals, in particular known toxic and carcinogenic metals, were delivered by weapons producing amputations, body charring, burns, and were associated with WP burns, all injuries without fragments detectable in the body of the victims.

We show metal presence in fragment-free wounds, and a specific metal signature for each class of injury. We also show that metal was detected in a decreasing gradient of concentration from the "hit" side towards the other adjacent tissues.

We have been examining samples not easily available, which posed the issue of limited numbers. Our samples had been "unusually treated" with metals, both in terms of pressure, temperature and directionality of the spread, which may cause uneven distribution over the wound, and the biopsies had to be dissected further in smaller fragments for analysis; both these facts potentially increase the heterogeneity that can be expected among fragments, which we found.

Nonetheless we describe a consistent pattern of presence/absence of each metal in different wounds, a fact to which, for simplicity, we refer as the "metal signature" for each class of injury.

Thus, the presence of metals in unusually high amounts in biopsies from wounds without fragments is taken as proof of the fact that these metals were delivered by the weapon. The decreasing gradient of metal content from the wound site to more distal tissues agrees with the assumption of topic targeting by weapons carrying the metals. The regularity in the association of "specific metal signature" to a particular class of injury, supports unequivocally the validity of associating a class of injury to a kind of metal-delivering weapon.

The inclusion in the study of Gazeans with different kinds of injuries and the finding of different specific metal signatures, eliminates also any doubt of intrinsic differences in metal in the tissues of Gazeans versus controls, eventually due to environmental factors.

Most of the metals detected in many fold amounts in excess over the control in the biopsies of the victims have lethal and acute intoxicating effects (e.g. As, Ba, Al, Hg) and cause chronic pathologies in time (Al, Pb, Hg, Cd, As), including mental, reproductive, lung, skin and kidney diseases [14,15].

In each class of injury were detected metals carcinogenic for humans (As, Cd, Hg, U, Cr (if CrVI), Ni (if oxide and sulfate)), or possible carcinogenic Co, V (if V_2_O_5_), Cu and Ni (if alloys)), or metals fetotoxic (i.e. genotoxic or/and teratogenic or/and impairing fetal development).

Some metals exert their effects in humans also via epidermal adsorption as Al and Hg, and others by os, as Mn, U, Cr (if VI) in human, and, in experimental animals, Cd, As, Co, Li, V, Mn, Zn and Cu.

In addition, U, Al, Ba, As, Cd, CrVI, Co, Cu, Pb, Hg, Ni, Sn and V behave as metalloestrogens, affecting sex hormones and glucocorticoid pathways, and interfering in sex determination, fertility and reproduction [16-26]. Toxic and carcinogenic metals are capable to interfere at the molecular level also with oxidative stress pathways and with DNA duplication and repair [25,26].

There is little information available on the effects of embodiment or assumption of many metals at once, as occurring in the victims here.

In addition, our analysis does not discriminate the isotopic form, the chemical associations or the physical status in which the metals are present, and, since their toxicity may vary with these, further investigation is necessary in these directions.

All these facts make difficult to provide conclusions on the effects that can be expected on the injuried, who assumed them during explosion of some kind and carry the metals embedded in their tissues. Nonetheless, the victims as well as whoever was in the vicinity, for a radium of unknown size, must also have been exposed to inhale and swallow the hot and high-pressurized metals dispersed by the blast. Also, around the target of weapons might have been produced a more or less large area of contamination of the ground by metals.

The final risks ensuing from the metal dispersion to people not directly hit possibly varies according to radium of spread, the absolute amounts and the specific characteristics (including metal form and aggregations) of each of the metals present in each of the weapon causing injuries without fragments.

Due to the many unknown factors illustrated above, it is possible only very adventitiously to calculate the pathogenic load of the amounts of metal in excess detected in the biopsies. Nonetheless, we compared the concentration of metals in the biopsies of victims with the minimal risk level (MRL) reported by ATSDR [27], for the cases where this is available and applicable, and based on the assumption that the amount of metal embedded in wounds is the minimal absolute amount the victim might have assumed via inhalation. We calculated that the amounts assumed are, for all metals in all cases, higher than the MRL for acute exposures, and than the known cumulative MRL for chronic exposures. This has exception for only one of the determination for one sample for U, and for all the Cd levels. At this time, calculations are merely indicative and many facts still need to be investigated, as mentioned above.

Even the biopsies from WP burns contain, beside Al, a known component of the sectors that isolate the WP load of the ammunitions used in Gaza, also amounts of toxic metals, embedded deeply into the flesh of the burned. These are medium amounts of toxic and carcinogenic Cu, Sr, Ba, Co, Hg, U and Pb.

The main motivation to undertake this study was that the information sought could be utilized either to rule out metal implication in non fragmentation weapons or, if they were involved, to help designing remediation strategies for children and youths of reproductive age known to have been exposed directly or by proximity to the attacks, and/or presently living at ground level in the places where these attacks took place, where contamination may persist (our unpublished data) with consequent chronic assumption by people.

The potential of chelating compounds to alleviate the burden from retention of metals has been described, with its side effects [28]. Melatonin has emerged as a promising remedy for metal intoxication [29]: it protects against metal induced oxidative stress, is a natural oncostatic (in Al, Zn Pb intoxications) and is partial inhibitor of the genotoxicity by Cd, has anti estrogenic activity, and has no known side effects [30,31]. Its limitation is that does not promote elimination of the metals from kidney, which can be achieved by chelating agents.

On the basis of our findings, we suggest that is an urgent priority to obtain information on the possibility of ongoing human contamination. Data on the metal amounts in the soil in this territory, collected before 2006, were published [32] and could be taken as reference for eventual future soil contamination studies.

## Conclusions

We show that metals, in particular known toxic and carcinogenic metals, were delivered by "not fragmentation weapons" producing amputations, body charring, burns, and were associated with WP burns.

We show that in each class of injury were detected metals carcinogenic for human, possible carcinogenic, or fetotoxic (i.e. genotoxic or/and teratogenic or/and impairing fetal development), with acute intoxicating effects and active in time, causing pathologies. In all cases, these were in amounts higher than the MRL for acute exposures, and than the known cumulative MRL for chronic exposures.

We show that the presence of these metals in fragment-free wounds differs for each class of injury, identifying a "specific metal signature" for each causing weapon.

We are not aware of data of similar nature or impact published before.

The information we present, that a specific cluster of metals characterizes different class of injuries without fragments, might be useful also for specific countermeasures, of immediate application, on survivors from injuries.

Projects of study on environmental and human contamination and remediation strategies should be undertaken and made possible through the International Institutions committed to these studies.

## Abbreviations

ppb: parts per billion (μg/kg); MRL: minimal risk level; WP: white phosphorus; A: amputation injury; C: death with charred body, M: deep burn injury by WP; B: burn injury.

## Competing interests

The authors declare that they have no competing interests.

## Authors' contributions

This research was commonly projected and designed by PM with SS, NAS and NAS and was concerted in its details with the analytical experts, Mar.B. and Mau.B. SS, NAS and NAS chose the cases, attended to their clinical classification, collection of biopsies and their preservation, and recorded the images of victims. MB and MB were responsible for the analytical work, and for controlling the results obtained in the other 2 labs involved. UG entered the team at the stage of the analysis of the data and completely assumed his responsibility and introduced his specialized outlook for the treatment and significance of the data. Tissue identification and histological study, preparation of samples and responsibility for the "blindness" in the study, commissioning to external laboratories of additional analysis, the coordination of the work among all authors, and the drafting of the manuscript was done by PM. All the other authors are completely aware of the process and final outcome, read and approved the final manuscript.

## Pre-publication history

The pre-publication history for this paper can be accessed here:

http://www.biomedcentral.com/1472-698X/10/17/prepub
